# Severe Congenital Diaphragmatic Hernia With Trisomy 9: A Case Report and Review of the Literature

**DOI:** 10.7759/cureus.28395

**Published:** 2022-08-25

**Authors:** Kazuya Fuma, Tomomi Kotani, Noriyuki Nakamura, Takafumi Ushida, Hiroaki Kajiyama

**Affiliations:** 1 Obstetrics and Gynecology, Nagoya University Graduate School of Medicine, Nagoya, JPN; 2 Obstetrics and Gynecology, Nagoya University Hospital, Nagoya, JPN

**Keywords:** prenatal genetic testing, trisomy 9, fetal ultrasonography, congenital diaphragmatic hernia, amniocentesis

## Abstract

Congenital diaphragmatic hernia (CDH) is known to be complicated with various chromosomal abnormalities. However, the grade of pulmonary hypoplasia of CDH complicated by trisomy 9 is not known. This information is essential to the mother who has had a fetus with the same complication. We report a case of severe CDH with trisomy 9. The fetus had fetal growth restriction and multiple anomalies, including severe left CDH (observed/expected lung-to-head ratio 13.7%, liver-up, stomach grade 3 in Kitano classification), mild ventriculomegaly, low-set ear, rocker bottom, and single umbilical artery. Chromosomal test by amniocentesis showed a karyotype of 47,XX,+9. The neonate was born alive at 34 weeks but died 49 minutes after birth. In the literature review, this case and seven cases of complete trisomy 9 had CDH, and four of them were explained as "large" or "severe" CDH. In conclusion, trisomy 9 might be occasionally complicated by severe CDH.

## Introduction

Congenital diaphragmatic hernia (CDH) is a life-threatening condition, occurring in one per 4000 births. It impairs normal airway and pulmonary vascular development. Therefore, CDH is associated with a high risk of neonatal death due to respiratory failure and persistent pulmonary hypertension. Recently, fetoscopic endoluminal tracheal occlusion (FETO) has been reported to improve the survival rates of severe left-sided CDH [1,2]. The FETO is performed for patients with <25% of the observed/expected lung-to-head ratio (o/e LHR), measured by a sonographic scan, at 27^+0^-29^+6^ weeks of gestation. A fetus with o/e LHR <25% is categorized as "severe" and estimated at <25% of survival rate. The prenatal severity estimation for CDH has also been established by liver position and stomach position grading like the Kitano classification [3]. In addition, the survival rate of infants with CDH has been reported to be lower in patients with other malformations or chromosomal abnormalities (non-isolated CDH) than in isolated CDH [4]. Therefore, a survey on other anomalies is important for providing an estimated prognosis to parents in prenatal counseling. In addition, information on chromosomal abnormalities helps select therapeutic approaches. Various genetic factors, including chromosomal abnormalities, copy number variations (CNVs), and single gene abnormalities, have been reported to be associated with CDH.

Among chromosomal aneuploidies, trisomy 18 has the highest frequency of posterolateral (Bochdalek) hernias, and trisomy 21 has the highest frequency of anterior (Morgagni) hernias. Trisomy 9 has also been reported to have a high incidence of CDH. The previous review of the 31 literature cases of necropsy for trisomy 9 showed that the complication rate of CDH to trisomy 9 was 9.6% [5]. However, case reports of CDH associated with trisomy 9 were sporadic, and the information remains insufficient for prenatal counseling. Therefore, it is required to accumulate the data of CDH with trisomy 9, although it is infrequent. In this case report and literature review, we present a case of severe left CDH with complete trisomy 9 and review previous literature on similar cases.

## Case presentation

**Figure 1:**
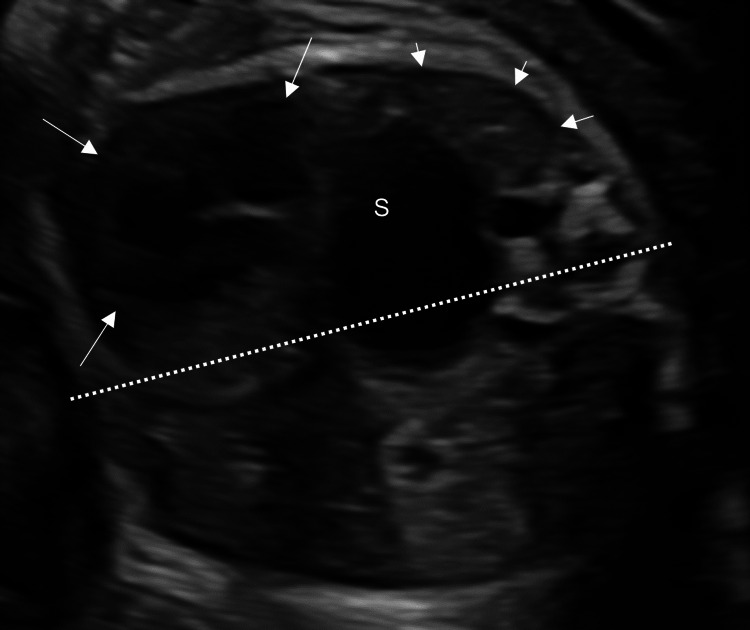
The 2D sonographic image of the fetal chest (horizontal section) Congenital diaphragmatic hernia with herniation of the stomach (S) more than halfway past the mid-line (dotted line). The heart (arrows) was shifted into the right thoracic cavity. The right lung was compressed and shrinking (arrowheads).

**Figure 2:**
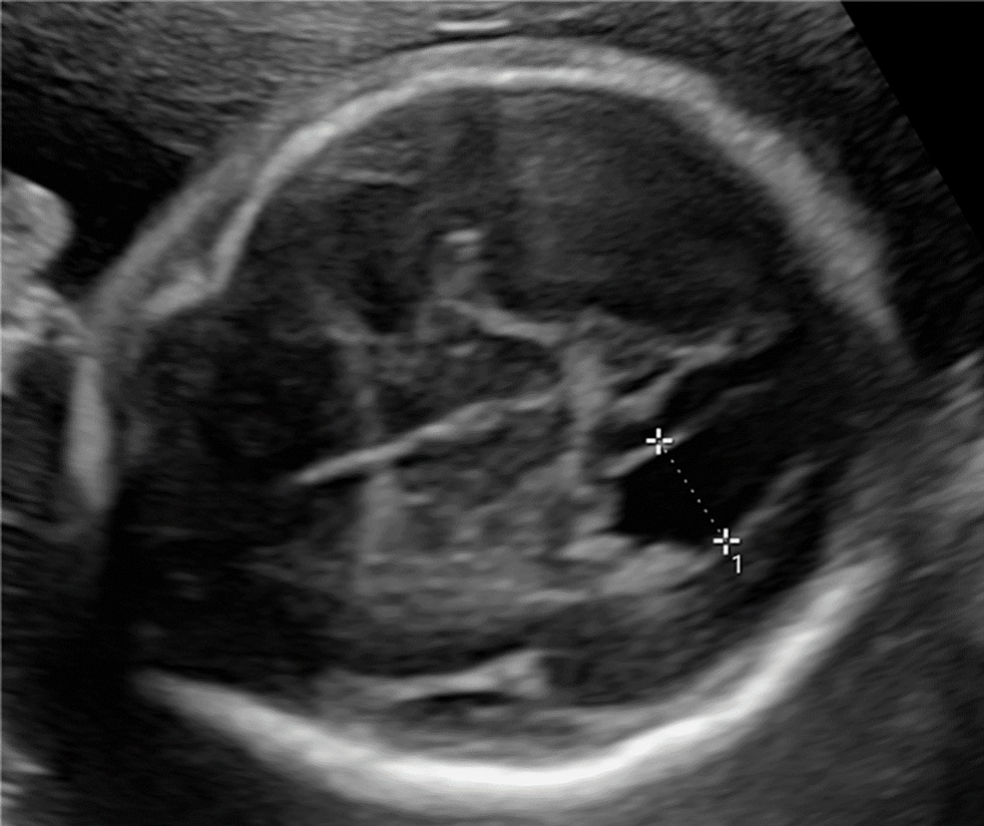
The 2D sonographic image of the fetal head (horizontal section) Mild enlarged atrial width (caliper, 11mm) is shown

**Figure 3:**
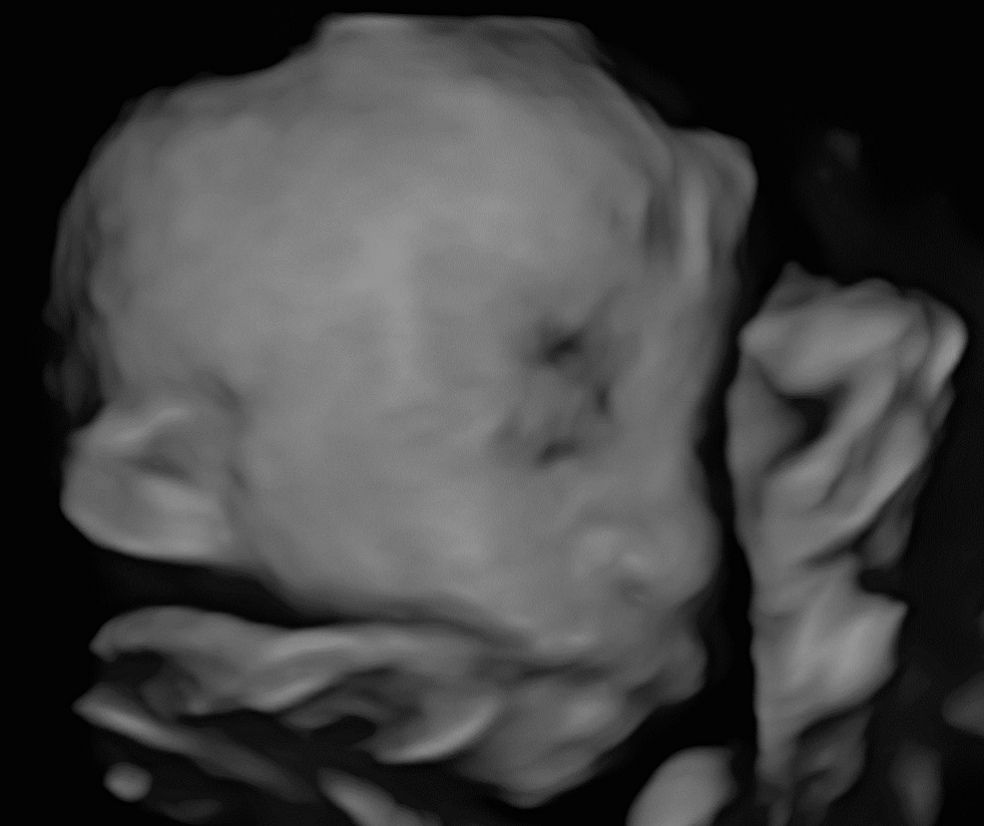
The 4D scan image showing a low-set ear

**Figure 4:**
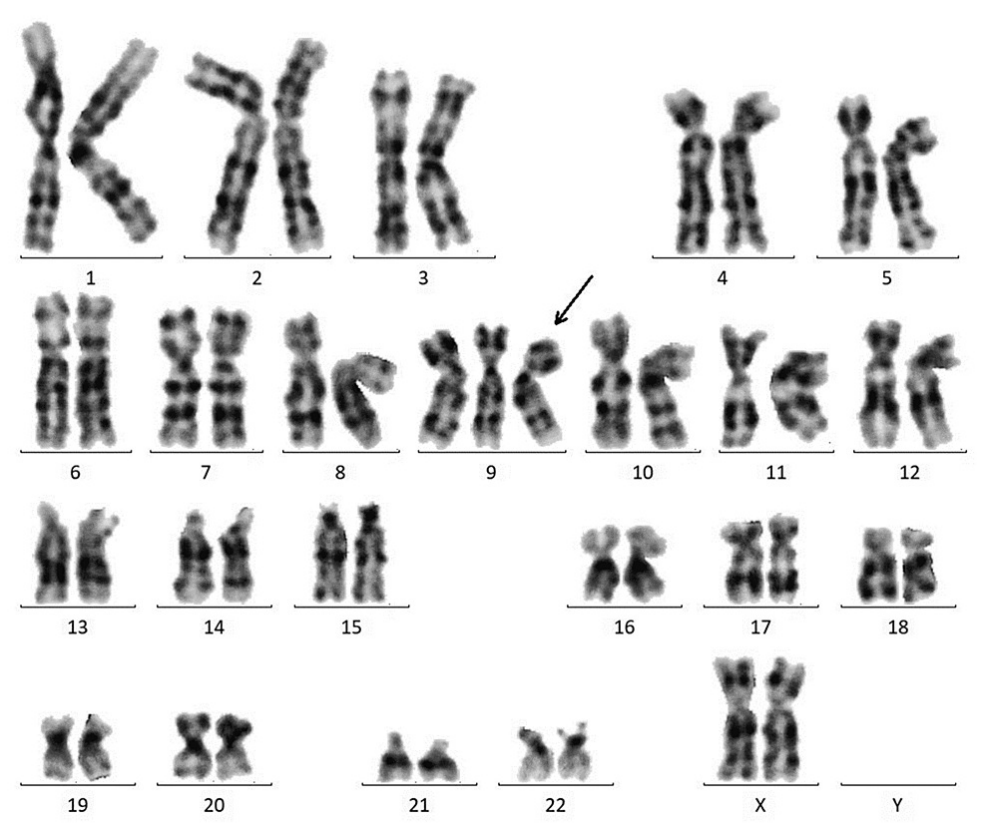
The result of G-banding by amniocentesis The arrow shows chromosome 9

A 34-year-old primipara woman was referred to our institution at 27 weeks of gestation with a fetal anomaly. Her partner was 31 years old. They had no history of medical conditions or medications except for pre-pregnancy multivitamins. At the first visit, we confirmed fetal growth restriction (−2.7SD), severe left CDH (o/e LHR 13.7%, liver-up, stomach grade 3 in the Kitano classification; more than half of the stomach herniated into the right chest, Figure 1), mild ventriculomegaly (atrial width 11mm, Figure 2), low-set ear (Figure 3), rocker bottom, and single umbilical artery. Artificial abortions after 22 weeks are not permitted in Japan. They hoped the best approach was to improve the survival rate of their baby as much as possible, although some genetic change had been suspected. After genetic counseling, we conducted amniocentesis for the chromosome test at the same time. Fluorescence in situ hybridization for chromosomes 13, 18, 21, X, and Y showed no aneuploidy. The deadline for FETO was approaching, and the incidence of aneuploidy was considered extremely low after excluding trisomy 13, 18, or 21 in the viable fetus until 28 weeks. At 28 weeks of gestation, she and her partner consulted on the advantages and adverse effects of FETO, including the information that was not covered by insurance in the institution where FETO is performed in Japan. After the discussion, they decided not to have a FETO for their baby because FETO might not improve the prognosis for CDH with multiple anomalies. At 29 weeks of gestation, the G-banding test was diagnosed as 47,XX,+9 (20/20cells, Figure 4). Magnetic resonance imaging at 32 weeks confirming severe CDH, which was consistent with the result of ultrasound evaluation, was also shared with a multidisciplinary team, including neonatologists, midwives, nurses, genetic counselors, and pediatric surgeons. She was hospitalized at 33 weeks of gestation due to a shortened cervix length, and the estimated fetal weight was 1403g (−2.8SD) at hospitalization. She and her partner hoped for aggressive treatment for their baby after birth. We provided the information on the expected prognosis based on the natural course of trisomy 9, low birth weight, and severity of CDH if treatment is provided to their baby in term delivery. However, at 34 weeks of gestation, uncontrollable uterine contraction began. Our multidisciplinary team again discussed the postnatal therapeutic approach with her and her partner, adding the negative impact of preterm birth on the prognosis. After the discussion, they decided on palliative care for her baby. She delivered a 1494g infant vaginally. The infant spent time immediately after birth with the parents without resuscitative procedures. The infant died 49 minutes after birth. We confirmed a low set ear and single umbilical artery in the infant. There were no findings of chorioamnionitis and polyhydramnios. Consent for pathological autopsy was not obtained.

## Discussion

Complete trisomy 9 is a lethal condition and primarily results in spontaneous abortion in the first trimester, therefore is rarely diagnosed prenatally or postnatally. Approximately 40 cases with trisomy 9 have been described to date. It is unclear which excess part of chromosome 9 is pathogenic, but one case report about tetrasomy 9p revealed CDH [6]; thus short arm of chromosome 9 may be associated with the pathological mechanism of CDH. Studies on de novo copy number variations (CNVs) have not yet shown an association between an excess of chromosome 9 and CDH. However, several genes, such as ABL1, TLN1, PLPP6, and NOTCH1 were reported to be associated with CDH as a de novo variant in chromosome 9 [7].

Congenital diaphragmatic hernia, fetal growth restriction, mild ventriculomegaly, low-set ear, single umbilical artery, and ventricular septal defect were detected in the present patient. All of them are consistent with previously reported phenotypes trisomy 9. Furthermore, the prenatal diagnosis was "severe" CDH with o/e LHR 13.7%, or "group III" with liver-up and grade 3 of stomach position in the Kitano classification. To our best knowledge, seven cases of complete trisomy 9 with CDH were identified (Table 1) [5,8-13]. Most of them were diagnosed as "large" or "severe" CDH in autopsy or prenatal sonographic scan. In addition, a complete hemi-aplasia and anterior-lateral defect were reported and considered Bochdalek and Morgagni holes, respectively. Thus, when CDH is complicated by trisomy 9, the hernia tends to be severe, and the defect position may be various. The present case was the first case of liveborn in the literature review.

**Table 1:** Summary of the literature on CDH complicated by trisomy 9 CDH: Congenital diaphragmatic hernia, NR: Not reported, TOP: Termination of pregnancy, AF: Amniotic fluid, CV: Chorionic villus, IUFD: Intrauterine fetal death, LHR: Lung-to-head ratio (severe CDH is LHR <1.0), o/e LHR: Observed/expected lung-to-head ratio (severe CDH is o/e LHR <25%).

Author (year)	Maternal age (years)	Gestational age at prenatal chromosome test (weeks)	Karyotype of chromosome test	Prenatal findings of CDH	Pathological findings of CDH	Outcomes
Frohlich (1982) [8]	45	NR	Trisomy 9	NR	Complete absence of the left hemidiaphragm, left CDH, with herniation of liver, spleen, stomach, and intestine.	TOP at 19 weeks
Suzumori et al. (2003) [9]	38	35	47,XY,+9 (AF, cord blood)	Large left CDH	NR	Stillbirth at 37 weeks
Sepulveda et al. (2003) [10]	40	12	47,XX,+9 (CV)	CDH	NR	IUFD at 31 weeks
Khoury-Collado et al. (2004) [11]	26	17 or more (NR)	47,XY,+9 (AF, skin, placenta, blood)	CDH	Large left CDH	TOP
Chen et al. (2004) [12]	25	16 or more (NR)	47,XX,+9 (AF, skin)	Left CDH	NR	TOP at 22 weeks
Ferreres et al. (2008) [5]	39	NR	47,XY,+9 (AF)	NR	Large anterolateral left CDH with herniation of spleen and left adrenal gland	TOP at 21 weeks
Serikawa et al. (2013) [13]	36	25	47,XY,+9 (AF)	Severe left CDH, LHR=0.83	NR	IUFD at 32 weeks
The present patient	34	27	47,XX,+9 (AF)	Severe left CDH, o/e LHR=13.7%, liver-up, stomach grade 3 by Kitano classification	NR	Spontaneous preterm delivery at 34 weeks, death in 49 minutes

Many patients of trisomy 9, including the present patient, had not been karyotyped in multiple organs. Therefore, mosaicism could not be excluded. In addition, two patients with trisomy 9 diagnosed using an amniotic fluid test were confirmed as trisomy 9 mosaicisms by postmortem examination [14,15]. Therefore, the present patient would also be mosaic trisomy 9, which may be the reason why she was liveborn.

In the present patient, CDH was complicated by trisomy 9, which was difficult to diagnose by only ultrasound findings. Therefore, a chromosome test should be considered before FETO in fatal patients.

## Conclusions

Trisomy 9 is occasionally complicated by CDH, which might be severe. The pathogenesis of severe CDH might be related to chromosome 9, although further research is needed. A prenatal chromosome test may also be useful in the postnatal management of CDH complicated by other anomalies.
